# Hybrid PET/MR metabolic imaging of the reperfused infarct - new biology, future directions

**DOI:** 10.1186/1532-429X-17-S1-O41

**Published:** 2015-02-03

**Authors:** Steven K White, Heerajnarain Bulluck, Georg M Frohlich, Steven G Casson, Celia O'Meara, Ayla C Newton, Peter J Weale, Ming Young S Wan, James Moon, Ashley Groves, Leon J Menezes, Derek J Hausenloy

**Affiliations:** 1 The Hatter Cardiovascular Institute, London, UK; 2 Independent Researcher, London, UK; 3 UCL Institute of Nuclear Medicine, London, UK; 4 Siemens Healthcare, Frimley, UK; 5 The Heart Hospital, London, UK

## Background

Multi-modality interrogation of myocardial health and disease using fused Positron Emission Tomography (PET) and magnetic resonance (MR) data has been limited by the need for separate scans, and complex and potentially imprecise co-registration of the individually-acquired PET and MR images. We sought to explore the potential of hybrid simultaneous PET/MR (hs-PET/MR) imaging to precisely localize changes in cardiac metabolism in areas of acute myocardial infarction (MI) and the area-at-risk (AAR) in acute ST-segment elevation myocardial infarction (STEMI) patients reperfused by primary percutaneous coronary intervention (PPCI).

## Methods

Hs-PET/MR (3Tesla Bio-graph mMR, Siemens) cardiac imaging was performed in 21 STEMI patients within 10 days of PPCI. A standard CMR protocol, included: localisers, functional cine imaging, with T2 mapping (WIP #699; Siemens Healthcare) and late gadolinium enhancement to delineate the AAR, and area of infarction respectively. Simultaneously, ^18^F-fluorodeoxyglucose (FDG) PET metabolic imaging was acquired, following patient preparation (fasting, oral glucose load, iv insulin). Matched short-axis slices of each modality: LGE, T2 maps, FDG, covering the entire left ventricle were analysed by 2 blinded investigators using 3 segmentation methods (in-house macro, ImageJ): manual; Otsu; 2 standard deviation (2SD) thresholding. 2 further investigators analysed respective coronary angiograms to derive BARI and APPROACH angiography jeopardy scores, both CMR-independent estimates of AAR size.

## Results

**Figure 1:**
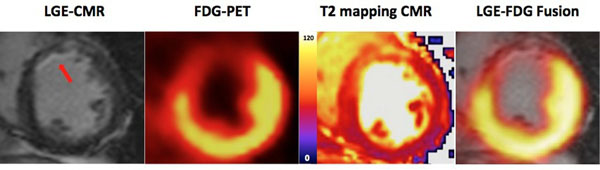
Multi-modality imaging of Subendocardial MI (arrows) with significant myocardial salvage. The area of reduced myocardial FDG uptake and edema on T2 mapping MR imaging (the AAR) extends transmurally and radially beyond the infarcted area (LGE, MR).

**Figure 2:**
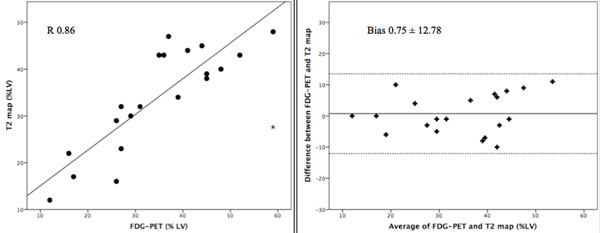
The AAR by FDG-PET imaging versus T2-mapping MR. There was excellent correlation and agreement between FDG-PET and the T2-mapping AAR (R=0.86; P<0.001; Bias 0.75±12.78%: no difference between their variances, P = 0.188).

The AAR delineated by T2-mapping correlated significantly with both the BARI (R=0.86;P<0.001) and APPROACH (R=0.80;P<0.001) scores, thereby independently validating this technique at 3T. In all patients myocardial FDG uptake was reduced in areas of MI (LGE). The total volume of myocardium with reduced FDG uptake was larger than the measured MI size (% left ventricle volume [mean±SD]: 35±12 versus 22±12; P<0.001), and was similar in size to the AAR measured by T2-mapping (% left ventricle volume [mean±SD]: 34±11 versus 35±12; P>0.05, Fig 1), with good correlation and low bias (R=0.86, bias 0.75±12.78%, Fig 2). Unexpectedly, we observed reduced FDG uptake in remote normal myocardium associated with microvascular obstruction and angiographic ‘no-reflow' (TIMI 0) in the infarct related artery.

## Conclusions

PET/MR FDG imaging of all zones of the acutely reperfused MI is feasible and adds complementary information. **Observations of new biology**: 1. Areas of reduced glucose metabolism occur in infarcted zones, but the areas are larger and match the AAR 2. Reduced metabolism in remote normal myocardium may represent a previously unidentified phenomenon, and requires further investigation. **Future directions**: Hybrid simultaneous cardiac PET/MR employing metabolism and/or novel tracers may advance pathophysiological insight into the acutely reperfused infarct, its sequelae, and the post-MI remodelled heart.

## Funding

This research study was funded by the British Heart Foundation (grant number FS/10/039/28270), the Rosetrees Trust, and was also supported by the National Institute for Health Research University College London Hospitals Biomedical Research Centre. SKW is supported by British Heart Foundation Clinical Research Training Fellowship (grant number FS/10/72/28568).

